# Deep iterative vessel segmentation in OCT angiography

**DOI:** 10.1364/BOE.384919

**Published:** 2020-04-10

**Authors:** Theodoros Pissas, Edward Bloch, M. Jorge Cardoso, Blanca Flores, Odysseas Georgiadis, Sepehr Jalali, Claudio Ravasio, Danail Stoyanov, Lyndon Da Cruz, Christos Bergeles

**Affiliations:** 1 School of Biomedical Engineering & Imaging Sciences, King’s College London, SE1 7EU, London, UK; 2 Wellcome/EPSRC Centre for Interventional and Surgical Sciences, University College London, W1W 7TS, London, UK; 3 Moorfields Eye Hospital, EC1V 2PD, London, UK; 4 Institute of Ophthalmology, University College London, EC1V 9EL, London, UK; 5 equal contribution; 6 theodoros.pissas.17@ucl.ac.uk

## Abstract

This paper addresses retinal vessel segmentation on *optical coherence tomography angiography* (OCT-A) images of the human retina. Our approach is motivated by the need for high precision image-guided delivery of regenerative therapies in vitreo-retinal surgery. OCT-A visualizes macular vasculature, the main landmark of the surgically targeted area, at a level of detail and spatial extent unattainable by other imaging modalities. Thus, automatic extraction of detailed vessel maps can ultimately inform surgical planning. We address the task of delineation of the *Superficial Vascular Plexus* in 2D Maximum Intensity Projections (MIP) of OCT-A using convolutional neural networks that iteratively refine the quality of the produced vessel segmentations. We demonstrate that the proposed approach compares favourably to alternative network baselines and graph-based methodologies through extensive experimental analysis, using data collected from 50 subjects, including both individuals that underwent surgery for structural macular abnormalities and healthy subjects. Additionally, we demonstrate generalization to 3D segmentation and narrower field-of-view OCT-A. In the future, the extracted vessel maps will be leveraged for surgical planning and semi-automated intraoperative navigation in vitreo-retinal surgery.

## 1. Introduction

Retinal vessels constitute the most salient anatomical landmark of the fundus and, as such, are commonly utilized as a biomarker for pathologies such as hypertensive and diabetic retinopathy [1–3]. Modern imaging systems produce rich visualizations of retinal vasculature, providing a basis for increasingly detailed automatically segmented vessel maps.

Vitreo-retinal (VR) surgery is currently performed manually, via small-gauge incisions in the eye through which tools as small as 0.2 mm are inserted. The surgeon uses a biomicroscopic viewing system to afford stereoscopic cues and identify anatomical features at the vitreo-retinal interface. This level of accuracy is sufficient to yield high success rates in current management of conditions such as epiretinal membranes and macular holes. However, emergent treatments in the form of cellular and gene-based therapies, which require precise delivery to specific retinal layers, challenge the current constraints of manual surgical precision [4,5]. Advancements in the development of robotics are likely to provide novel means of semi-automated delivery of epi-, intra- and sub-retinal therapies [5]. In order for these systems to operate safely and effectively, they will require highly-precise sensory navigation mechanisms, for which automated identification of retinal vasculature will prove invaluable.

**Fig. 1.**
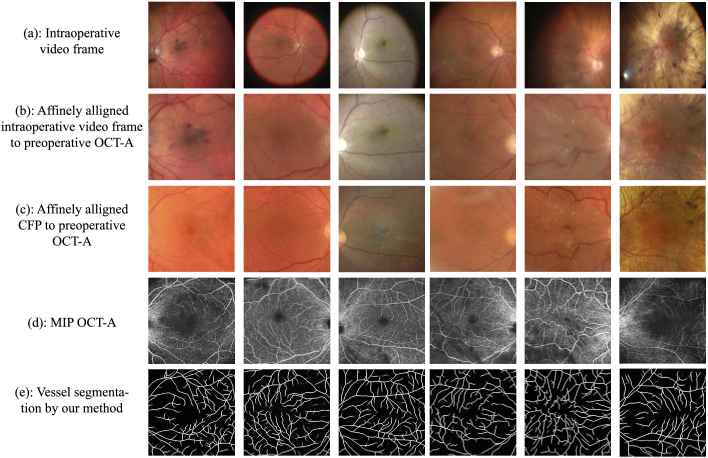
OCT-A vs Preoperative and Intraoperative Imaging: For several subjects with retinal pathology we present: (a) An Intraoperative Video Frame (IVF) captured during vitreo-retinal surgery (b) IVF affinely aligned to OCT-A (c) a preoperative CFP affinely aligned to OCT-A, (d) the OCT-A and (e) the vessel segmentation obtained by our vessel segmentation method. In all cases, OCT-A visualizes the maximum level of vasculature detail around the surgical RoI, the macula, where both CFPs and IVFs tend to provide blurry information and are susceptible to subretinal pathology, such as choroidal pigmentation (last column), that impacts retinal vessel visibility.

In VR surgery, the primary region of interest (RoI) is frequently the macula, *i.e.* the central retinal area, which is bound by temporal retinal vessels and contains the fovea, responsible for high-acuity central vision. Despite the high density of retinal vasculature within the macula, there is a relative paucity of information that can be resolved from color fundus imaging of this region due to small vessel caliber. To address this, we utilize Optical Coherence Tomography-Angiography (OCT-A) scans that attain a superior level of detail in comparison to other pre-operative and intra-operative imaging, especially around the surgical RoI, as exemplified by Fig. 1. Our goal is to produce a map of the vessels in the vicinity of the macula, with the maximal attainable detail, preoperatively. This constitutes a key first step towards intra-operatively leveraging this rich information about the surgical workspace, which can only be visualized and extracted pre-operatively.

### 1.1 OCT-A vs other preoperative modalities

OCT-A is a relatively new, non-invasive, rapidly acquired imaging modality derived from the amplitude decorrelation and phase variance between sequential OCT B-scans [6], resulting in a static 3D blood motion map with very high resolutions across all dimensions albeit for a limited field-of-view (FoV) of up to 8mm by 8mm. The current gold-standard for retinal vasculature visualization is Fundus Fluorescein Angiography (FFA), which allows dynamic high contrast visualization of blood flow, offers a superior FoV and is less susceptible to artefacts. However, FFA is invasive, requiring the administration of an intravenous contrast agent with potential systemic adverse effects (including anaphylaxis [7,8]), and has a much longer acquisition time than OCT-A (at least 20 minutes compared to 10 seconds [9]). FFA is, however, able to demonstrate dynamic vascular processes, such as leakage and staining, which cannot be interpreted using OCT-A. Clinically, OCT-A is able to visualize vascular abnormalities such as choroidal neovascular membranes and capillary non-perfusion in great detail, with comparable diagnostic yield to FFA. But, unlike FFA, it is also able to provide 3D information about the level of the pathology, enhancing understanding of retinal vascular disease and guiding treatment approaches and responses. Consequently, OCT-A is becoming increasingly popular for routine clinical use and, importantly for our work, it allows for data collection with minimal psychological and physical burden on participants. An alternative non-invasive method of visualizing the retinal vasculature would be a preoperatively acquired Color Fundus Photograph (CFP) delivering a FoV of 45° that corresponds to roughly 20% of the retina. Despite the enlarged FoV of CFP, OCT-A offers superior level of vascular details especially around the macula as shown in Fig. 1.

**Fig. 2.**
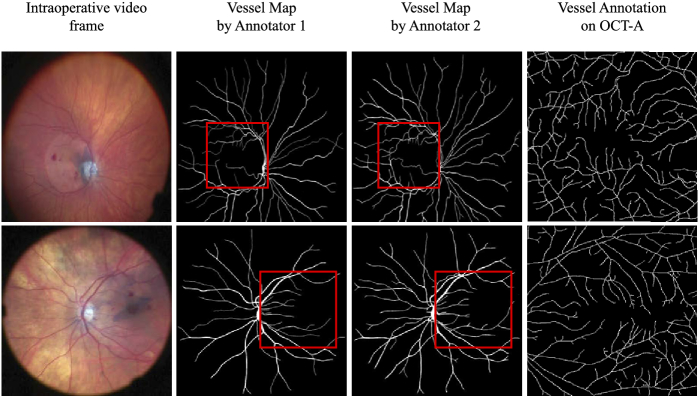
Intraoperative vs OCT-A Vessel Visibility: Vessel map annotations by two expert clinicians on intraperative video frames revealed that in the vicinity of the macula (outlined in red) they are unable to detect the level of vasculature details that can be reliably annotated on OCT-A.

This discrepancy is also supported by our observation that expert clinicians tended to only detect the bigger vessels, further from the fovea, when annotating preoperative CFPs or intraoperative frames, while the same process on OCT-A reveals significantly finer details, as shown in Fig. 2. This hints on the complementarity of information conveyed by the two modalities, the fusion of which is likely to produce superior retinal feature localization during surgery. It is therefore anticipated that vascular information from both pre-operative CFPs and OCT-A can be used to enhance intra-operative features and improve intraocular navigation and orientation for precise therapeutic delivery.

### 1.2 State-of-the-art retinal vessel segmentation

Vessel segmentation falls within the scope of the more general problem of curvilinear structure delineation in 2D or 3D images. In this section, we summarize methods that have been applied on retinal vessel segmentation. The majority of reported methods have been evaluated on 2D CFPs from the publicly available datasets such as DRIVE and STARE [10,11]. Prior to deep learning, methods consisted of either a *hand-crafted* feature extraction step [10,12,13] or the application vessel enhancement filters [14–16], followed by either a supervised classifier or heuristic post-processing. Subsequent methods attempted to automate feature extraction via supervised learning of filters learned through sparse coding [17], Gradient Boosting [18], Conditional Random Fields (CRF) [19] or regression of the vessel map’s distance transform [20]. Several other works formulate the problem of curvilinear structure segmentation as a two step process: first generating an overcomplete graph via tubularity filtering [15] and computation of minimal-cost paths between highly tubular points [21,22], followed by graph pruning that results in a subgraph that corresponds to the vessel map. The pruning step is treated as an optimization problem coupled with vessel tree local geometry priors [23] or path-classifiers trained to score small parts of the graph to facilitate the convergence of the optimization algorithm [24].

Deep learning was first utilized in [25], where feature maps from multiple layers of a Convolutional Neural Network (CNN), pretrained for large-scale image classification, are combined through additional convolutional layers and fine-tuned to produce vessel segmentations. This idea was extended through the use of CRFs [26] to model non-local dependencies in the image.

Few publications on retinal vessel segmentation in OCT-A exist, which can be attributed to OCT-A being a recently introduced imaging modality and the complete lack of publicly available datasets for method comparisons. In [27], a form of Markov Random Field was applied on OCT-A scans of healthy subjects and subjects with Diabetic Retinopathy. In [28], a CNN operating on small overlapping 2D patches of narrow FoV OCT-A images, in a sliding window fashion, was used to classify center pixels as vessels or background with the model being evaluated on 6 healthy volunteers.

**Fig. 3.**
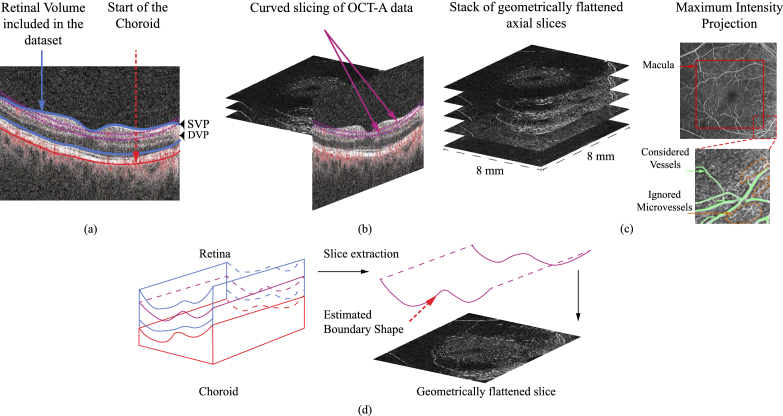
OCT-A data overview: (a) Retina cross section: outlined in blue is the volume corresponding to the slices used in the dataset. They span the space from the retinal surface (upper limit of the blue line) to the start of the choroid (outlined in red) where the Superficial and Deep Vascular Plexuses are located. (b) The imaging device produces geometrically flattened slices that correspond to curved slices of the retina’s cross-section. (c) Maximum Intensity Projection is performed on the extracted stack of geometrically flattened slices along the axis vertical to the plane of the slices. Outlined in red is the (approximate) location of the macula around which the scans are centered. The zoomed-in patch depicts (in green) vessels that are considered by our models and areas (in orange) where microvessels are likely to be located, which however cannot be delineated reliably and the models learn to ignore. (d) The imaging device locates the limiting surface between the retinal layers (blue space) and the choroid (red space) allowing us to access geometrically flattened slices thus separating chroroidal and retinal layers.

Our approach differs from these works in several aspects. Contrary to [28], we use fully convolutional networks to segment the whole image with each feed-forward pass and employ OCT-A images of an expanded FoV, thus encompassing more context in the vicinity of the macula rather than just the fovea. Contrary to [27], we choose to train and test our models on the task of segmenting all vasculature within the imaged space but omit microvessels (see Fig. 3(c)) that may be visible but cannot be reliably annotated due to the inherent difficulty in inferring their shape and connectivity, especially in the 8mm by 8mm scans. Finally, we believe that works that address the task foveal avascular zone (FAZ) quantification in OCT-A [29] are complimentary to our vessel segmentation method and potentially there exists a synergy between the two tasks due to their common spatial and functional support.

### 1.3 Contributions

This paper demonstrates that Recurrent Fully Convolutional Neural Networks trained with a *perceptual loss* are the most effective solution for precise and accurate vessel segmentation in OCT-A images; our work builds on the contributions of [30–33] and [32,34,35]. Our conclusion is supported by extensive experimental comparison of CNN architectures on a newly collected, challenging dataset, the first with manual annotations of vessels (more than existing datasets with annotated retinal vessels in CFPs) in 8mm×8mm OCT-A, comprising subjects that underwent VR surgery for structural macular abnormalities. We aim to make this dataset public, through our collaboration with INSIGHT - the UK’s Health Data Research Hub for Eye Health. Further, we demonstrate that our network can generalize to 3 mm×3 mm OCT-A scans that provide a higher resolution of the macular area but for a narrower FoV. Finally, we demonstrate the recovery of 3D vascular trees from OCT-A volumes. To the best of our knowledge, this gives rise to the first 3D visualization of retinal vasculature derived from OCT-A.

To facilitate computational retinal image understanding research and boost potential use by practitioners interested in aligned domains, such as diagnostics, the source code of our method as well as trained models will be available online at https://github.com/RViMLab/BOE2020-OCTA-vessel-segmentation.

## 2. Materials and methods

This section outlines the process of creating the OCT-A dataset. We provide details on the location of the 3D retinal space on which we focus on: namely the space between the vitreo-retinal interface and the choroid. Figure 3 provides an overview of the data extraction process.

### 2.1 Dataset collection and preparation

The study was conducted in accordance with the tenets of the Declaration of Helsinki (1983 Revision) and the applicable regulatory requirements. After approval of the study and its procedures by the ethics committees of Moorfields Eye Hospital, London, United Kingdom, informed consent was obtained from all participating subjects prior to enrollment.

OCT-A Scans were collected from 50 patients using Zeiss Cirrus 5000 with Angioplex. Motivated by our VR surgery-related application, we selected participants that were referred to surgery due to structural macular abnormalities. The distribution of pathologies represented in our dataset is summarized in Table 1.

**Table 1.** Distribution of types of pathology in the dataset

Pathology	Macular hole	Epiretinal membrane	Choroideremia	Optic disk pit maculopathy	Floaters	Asteroid hyalosis	Disclocated IOL	Healthy
**Subjects**	16	9	7	5	3	1	1	8

Figure 3(a) demonstrates the location of the region of interest on the cross-section of the retina. The imaging device allows us to view the data as a series of *geometrically flattened* slices of the 3D volume, allowing separate viewing of the otherwise curved retinal and choroidal layers. More specifically, it allows curved slicing of the OCT-A data, where the curve shape is an estimate of the boundary between the choroid and the retina as outlined by 3(b). We use the term flattened to denote that the curvature of those slices is factored out as they are viewed as planar images, as shown in 3(d). This view is the one used in clinical practice and we leverage it to manually extract the set of contiguous slices that correspond to the retina, each corresponding to a surface of 8 mm×8 mm. The thickness of the retina is patient- and disease- specific, and therefore the number of extracted slices may vary. The resulting extracted volume spans the Superficial (SVP) and Deep (DVP) Vascular Plexuses [36]. Finally, the resulting stack of axial slices is projected to 2D via *Maximum Intensity Projection* (referred as MIP) along the axis vertical to the plane of the slices, as illustrated in Fig. 3(c).

The MIP of the set of geometrically flattened slices serves as input to all 2D vessel segmentation methods explored in this work. All MIPs have a pixel count of 416×416 representing a FoV of 8 mm×8 mm, implying a resolution of approximately 19μm. Vessel centrelines on each of the 50 MIP images were manually annotated using the Vampire software, available online at vampire.computing.dundee.ac.uk. Centreline extraction was preferred to full-width segmentations because consistent full-width annotation was difficult to attain due to fading contrast away from the centreline, in addition to the width of the vessels rarely being larger than a couple of pixels.

The centrelines were annotated by a post-graduate researcher that was trained and advised by expert clinicians with regards to OCT-A interpretation. A clinical expert annotated a set of images to produce a metric for inter-rater variability, while a set of images were annotated twice to estimate intra-rated variability. An annotator was allowed to zoom in and out of the image as much as required to increase delineation confidence. Further, he/she was allowed to extrapolate vessel/branch connectivity by examining the region surrounding vessels corrupted at pixel-level by scanning artefacts. The MIPs also contain microvasculature that is essentially filling up most of the space between bigger vessels. When blood flow in these microvessels is captured in the OCT-A images, the regions where these are is brighter 3.c. Their shape, however, cannot be reliably inferred even by a human observer and their presence is not clinically important to our overarching aim, which is to provide a vessel map for guiding VR surgical interventions.

### 2.2 Problem formulation and notation

Vessel segmentation is formulated as a set of binary classification problems, one for each pixel $x_{i}$ of the input image $X\in R^{H\cdot W}$, with H,W being the height, and width of the input image, respectively. For each pixel $x_{i}$ we predict the posterior probability $\hat{y_{i}}=P(y_{i}=1|X)$ that it lies on a vessel. The ground-truth labeling for each pixel is denoted by $y_{i}\in \{0,1\}$ and has a value of 1 if the pixel belongs to the vessel and 0 if it belongs to the background. Finally, $f(X;\theta ):R^{H\cdot W}\mapsto {\left[\;0,1\right]}^{H\cdot W}\;$ denotes the function implemented by a convolutional neural network, which is parameterized by weights θ.

### 2.3 Base network

**Fig. 4.**
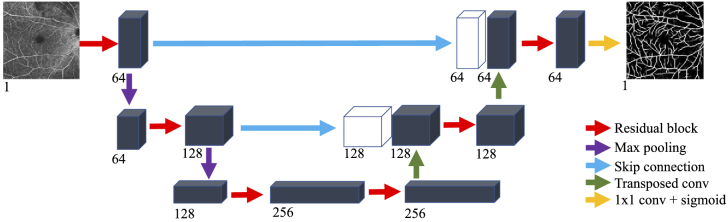
Schematic representation of the architecture of the base-network as described in Sec. 2.3. The base-network follows the architecture paradigm of UNet. The number below any tensor denotes the number of feature maps at that stage of the network.

In this work, f is implemented by a *UNet* [31] with a modified architecture. *UNet*-like networks have exhibited excellent performance in natural image segmentation [37,38], generation tasks [39], and medical image segmentation [40]. Importantly, these networks naturally preserve the input’s resolution to the output. Our modified *UNet* is herein termed *base network* and is schematically depicted in Fig. 4.

Contrary to the original UNet architecture, we employ residual blocks, as described in [41], instead of simple convolutional layers at each resolution. Each convolution in every residual block uses a stride of 1 and zero padding such that the resolution of the output feature maps is equal to the resolution of the input feature maps.

The *encoder* part of the network is composed of 3 residual blocks, with the first two being followed by max pooling that subsamples the incoming feature maps by a factor of 2. At each subsequent residual block, the number of filter kernels (and, thus, feature maps) at all blocks’ convolution layers is double that of the previous block’s.

The *decoder* part of the network also consists of 3 residual blocks, with the first two being followed by a transposed convolution layer that increases the resolution of the feature maps by a factor of 2. The last residual block, which has the same spatial resolution as the input, is followed by a simple convolution with a 1×1 kernel.

ReLU non-linearities are used throughout the network except for the linear output of the last convolutional layer of the decoder, where the sigmoid function is applied element-wise to produce the final confidence scores in [0,1].

### 2.4 Iterative refinement

Most semantic segmentation networks produce their final output in a single forward inference pass [30,31,42] as does the base-network described in the previous section. For the delineation of fine structures in noisy images, such as OCT-A, the single pass constraint leads to false positives and topological inaccuracies, *e.g.* holes that break the continuity of vessels. We relax this constraint and seek to improve the quality of delineations by applying and evaluating iterative refinement using two different approaches. In both cases we utilize the UNet base network.

The first approach employs a *Stacked Hourglass Network* (SHN), proposed in [38], that is composed of distinct cascaded UNet modules. The SHN, using multiple encoder-decoder modules, can learn to infer vessel location in a coarse-to-fine manner by feeding intermediate predictions to subsequent modules. Additionally, concatenating intermediate predictions with the input image and feeding them to the subsequent module pushes it to learn to refine the result by attending to regions of the input image where vessels were previously detected.

**Fig. 5.**
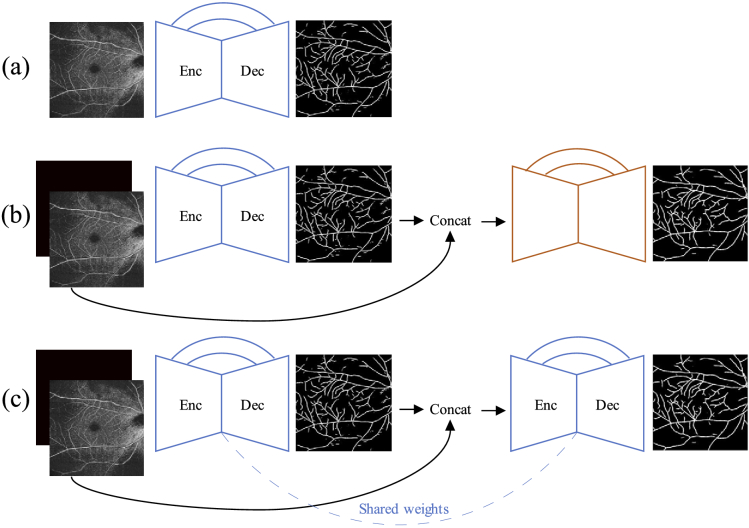
Considered CNN architectures: (a) UNet, (b) SHN, and (c) iUNet. For illustration purposes the SHN, and iUNet, are presented with 2 distinct UNet modules, and 2 iterations, respectively.

The second approach considered is based on the refinement method proposed in [32,33,43], where a single network, in our case the UNet-like base networks of Sec. 2.3, is employed in a recurrent manner, by recurrently feeding intermediate predictions in the network to obtain refined predictions (iUNet). The key element of this model design is that the number of parameters (model weights), stays constant regardless of the number of refinement iterations performed. Moreover, the end-to-end model is directly guided to learn to correct its own mistakes, whereas each module of the SHN learns to resolve mistakes of the modules that preceded it. The two approaches are illustrated in Fig. 5.

### 2.5 Loss functions

This section describes the loss functions that were evaluated to conclude as to the combination that achieves the most promising OCT-A segmentation results. Preliminary experiments suggested that using the loss of [44], which is balanced according to class frequency, instead of simple cross-entropy loss stabilizes training and improves performance as has also been demonstrated in [25]. Using the notation of Sec. 2.2, we have:

(1) $$\begin{array}{rl} & L_{bce}=-\beta \sum_{i\in Y^{+}}\log(P(y_{i}=1|X;\theta )-(1-\beta )\sum_{i\in Y^{-}}\log(P(y_{i}=0|X;\theta ), \end{array}$$

where $\beta =|Y^{-}|/|Y|$ and $1-\beta =|Y^{+}|/|Y|$, $Y^{-}$, and $Y^{+}$ are the sets of pixels that lie on vessels, and background, respectively, and ∣.∣ denotes a set’s cardinality.

In addition to the classification loss, we also evaluated the effect of the perceptual loss [34,35] to penalize topological inaccuracies in the network’s predictions, as proposed in [32]. This loss term utilizes the VGG19 [45] network pretrained on Imagenet [46] to extract feature maps from the ground truth segmentation and the output segmentation. The loss term is then the $L_{2}$ norm of the difference between those feature maps, and is referred to as topological or perceptual loss. More specifically:

(2) $$L_{topo}=\sum_{n=1}^{N}\frac{\mu_{n}}{W_{n}H_{n}C_{n}}\sum_{c=1}^{C_{n}}{\left\|F_{n}^{c}(f(X;\theta ))-F_{n}^{c}(Y)\right\|}_{2}^{2},$$

where $F_{n}^{c}$ is the c-th channel (from a total of $C_{n}$ channels) of the $W_{n}\times H_{n}$ feature maps extracted at the n-th layer (from the total of N layers) of the VGG19 that are used. In practice, we utilize N=3, where the included feature maps are the ReLU activations of $conv_{12},conv_{22},conv_{34}$ of the VGG19 network. The weighing factor $\mu_{n}$ controls the importance of the n-th layer’s feature maps. Finally, the cross-entropy and topological losses are combined as follows:

(3) $$L_{comb}=L_{bce}+L_{topo}$$

It is noted that the two terms are weighted through factors $\mu_{n}$ of (2), contrary to [32] where a single scalar factor is used.

When training the SHN we compute the loss of (3) after each of its modules, using its respective prediction, and sum the resulting terms. This differs from the intermediate supervision proposed in [38], in that we augment each loss term with the perceptual loss term (3).

To train the iUNet, we again compute the loss of (3) after each iteration, and the final loss is the weighted sum of the resulting terms. Based on our observations, weighing loss terms originating from different base network iterations is critical as otherwise training may become unstable. We adopt the weighing of [32], which weighs later iterations higher while keeping the sum of the weights equal to 1. This scheme explicitly forces the network to learn to produce a coarse-to-fine segmentation. Specifically, the overall loss for the iUNet is:

(4) $$L_{iUnet}=\frac{2}{T(T+1)}\sum_{t=1}^{T}tL_{comb}^{\left(t\right)},$$

where T is the number of base network iterations. In our experiments, we set T=2,3,4,5, as using more iterations did not significantly improve performance. Finally, we note that contrary to [32] we train the models for all number iterations T in one go, instead of sequentially and separately optimizing for $T=1,2,\ldots ,T_{max}$. End-to-end training avoids the complexity of running T training stages.

## 3. Experiments and evaluation

This section describes the experimental protocol that was followed to train and evaluate the network-loss function combinations that were tested in search of the optimal one. Additionally, these experiments aim to identify the importance of different loss terms and network hyper-parameters on performance.

### 3.1 Evaluation metrics

To evaluate the performance of all methods, we compute the *Completeness*, *Correctness* and *Quality*, as introduced in [47]. Let a ground truth centerline be Y and the vessel-continuity-preserving skeletonized [48] binarized prediction (threshold = 0.5) of the algorithm under evaluation be $\hat{Y}$. Additionally, we denote the set of points of skeleton A that match a point of skeleton B as $\mu_{B}(A,\tau )=\{\alpha \in A|\exists \beta \in B:\left\|a-b\right\|\;<\;\tau \}$, where τ is a tolerance factor in pixels to acknowledge the unavoidable uncertainty entailed in delineating fine structures, such as blood vessels, occasionally merely a couple of pixels wide. Then:

(5a) $$\text{Completeness}=\frac{\mu_{\hat{Y}}(Y,\tau )}{|Y|},$$

(5b) $$\text{Correctness}=\frac{\mu_{Y}(\hat{Y},\tau )}{|\hat{Y}|},$$

(5c) $$\text{Quality}=\frac{\mu_{Y}(\hat{Y},\tau )}{|\hat{Y}|-\mu_{\hat{Y}}(Y,\tau )+|Y|}.$$

Conceptually, *Completeness*, and *Correctness* constitute a “relaxed” version of *Recall*, and *Precision*, respectively; *Quality* summarizes them into a single measure and can be considered a “relaxed” version of *Intersection over Union*. Moreover, we compute the *Precision-Recall break-even point* [49], denoted by *PR*. When Precision equals Recall, the PR corresponds to the F-score (or DICE). This metric is computed by pixel-to-pixel comparisons between the output of the network and the ground truth centerline dilated by 1 pixel.

To estimate inter-rater agreement, 10 images were annotated by a second annotator as well. We computed the Quality metric for one annotator against the other using different tolerance factors, namely τ=1,2,3, which resulted in ${\text{Q}}_{\text{rater-1},\tau =1}=0.3415$, ${\text{Q}}_{\text{rater-1},\tau =2}=0.8016$ and ${\text{Q}}_{\text{rater-1},\tau =3}=0.8253$, respectively. Effectively, τ=1 corresponds to not introducing any tolerance as the metric is computed on a discretized pixel grid, meaning that the euclidean distance between points is at least 1. This justifies the very low performance of rater-1 against rater-2. Reasonable agreement is obtained by introducing a tolerance of 2, which corresponds to allowing matched points to be at most direct neighbours on the pixel grid. Using this observation we fix the tolerance factor to τ=2 for every reported *Quality*, *Correctness*, and *Completeness*. We also estimated intra-rater agreement over 5 images, which resulted in ${\text{Q}}_{\text{intra},\tau =1}=0.3623$, ${\text{Q}}_{\text{intra},\tau =2}=0.8922$ and ${\text{Q}}_{\text{intra},\tau =3}=0.9358.$

### 3.2 Training details

We trained all models using the Adam optimizer [50] with a batch size of 2 and an initial learning rate of $10^{-4}$, decayed using inverse time decay scheduling with a rate of 0.5. As our ground truth annotations are vessel centerlines, we dilate them by 1 pixel to densify the supervision signal. During training, after each epoch, we evaluate the model on a validation set by computing the *Quality* metric after first thresholding at 0.5 and skeletonising the predicted probability maps. We train all models for up to a total of 6K steps, which corresponds to roughly 96 epochs. Preliminary experiments revealed that, as expected, models with more parameters required more training steps to converge to their maximum validation performance. Specifically, we experimentally found that 6k steps were enough for all models to reach their final validation performance. Following the *Early Stopping* paradigm, we stop training if for 10 consecutive epochs validation performance does not improve and if a minimum of 1K training steps have elapsed, with the goal of maintaining a balance between adequately fitting the training data and not implicitly overfitting the validation set.

### 3.3 Data augmentation

**Fig. 6.**
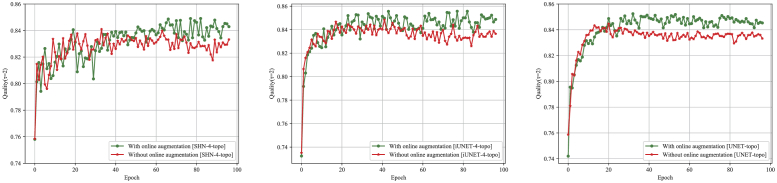
For all models, adding online data augmentation during training (described in 3.4) prevents overfitting by regularizing training while leading to higher validation Quality. The presented curves are computed when training and validating on the same cross-validation fold of the dataset, but this finding was consistent across folds.

Given the limited data available, in comparison with natural image datasets, data augmentation was essential for regularizing and inducing invariances to the learned model and avoiding over-fitting. We perform rotations by 90°,180°,270° and append the transformed images to the original training set. Rotating OCT-A images with naturally occurring horizontally oriented artefacts [51] produces vertically orientated artefacts that are not plausible. For the task we consider, however, this does not constitute a hindrance as our models will be trained with the more general requirement of ignoring both vertical and horizontal artefacts, and importantly on a variety of rotated, plausible vessel shapes. Prior to each training iteration, we perform scaling, brightness distortions and contrast distortions by factors uniformly sampled from [0.8,1.3],[0,0.2], and [0.75,1.25], respectively; deformation by randomly generated smoothed deformation fields as in [31]; random erasing of multiple small 4×4 input regions similar to [52]. Figure 6 demonstrates *Quality* evaluated on the validation set after each training epoch with and without on-line data augmentation. On-line data augmentation significantly limits over-fitting and allows the model to achieve higher maximum performance.

### 3.4 Experimental comparisons

We treat the base network, *i.e.* a single UNet, referred to as **unet**, with the architecture described in Sec. 2.3, trained with the loss of (1) as the baseline. Then, we trained the same UNet with the combined loss of (3), which is referred to as **unet-topo**. In all experiments with the loss of (3), we included the $conv_{12},conv_{22},conv_{34}$ feature maps of the VGG19 network with $\mu_{12}=10^{-2}$, $\mu_{22}=10^{-3}$, $\mu_{34}=10^{-4}$ as their respective weighing factors chosen experimentally as described in the appendix.

To compare training from scratch (as proposed here), with fine-tuning a Imagenet-pretrained model, we also train the network of [25], denoted as **DRIU**, which is based on a pretrained VGG16.

Furthermore, we trained the iUNet model using the loss of (4) but without the perceptual loss setting $\mu_{12},\mu_{22},\mu_{34}=0$ and for k=2,3,4 refinement iterations. The resulting models are termed **i-unet-*k***. Training these models with the perceptual loss term active gives **i-unet-*k*-topo**.

Similarly, we trained the SHN model with k=2,3,4,5 modules using the loss of (3) at each module’s output, denoted by **shn-*k*-topo**, and without the perceptual loss denoted by **shn-*k***. Finally, we ablate base-network depth by training **unet-topo**, **i-unet-4-topo** and **shn-4-topo** with a base-network of 5 (vs 3 used in all other cases) residual blocks in both encoder and decoder.

We used the data augmentation scheme of Sec. 3.3 and 4−fold cross validation for all experiments. Finally, to determine the statistical significance of differences observed between models, we conducted paired Wilcoxon signed-rank tests on the quality metric derived from individual subject segmentations obtained from the network trained with the fold for which the subject is in the held-out test set.

### 3.5 Cross validation and model selection

To select the optimal model-loss combination we employ 4*-fold cross-validation*. Specifically, we utilize stratified sampling to partition our data into 4 folds, each of which is composed of disjoint training, validation, testing sets. This ensures, that each pathology is represented in all 3 sets. As a result, we use 27,30,30,31 images in the training sets (respectively for each fold), 8 images in the validation sets and 15,12,12,11 images in the test sets (unseen during training). Testing sets of different folds are disjoint (i.e merging them gives us the whole dataset), meaning that each subject is in the testing set of only one fold, and is either in the training or validation sets of all other folds. As described in Sec. 3.3, via fixed rotations the final training set sizes are 108,120,120,124. The mean and the standard deviation of the evaluation metrics on the test set across the 4 folds is reported. We combine cross-validation with statistical significance tests to determine whether the observed differences in Quality between models can be attributed to random effects caused by the stochasticity of the training algorithm (Sec. 3.2, 3.3) and the choice of dataset fold, or are truly characterising the behaviour of the models.

## 4. Results

This section presents the results that were obtained by the experiments outlined in Sec. 3.4, concludes on the optimal model-loss function and on the importance of depth, refinement iterations and dataset size on delineation performance.

### 4.1 Quantitative evaluation and comparisons

Unsurprisingly, weakly supervised graph-based method (described in the appendix), is outperformed by all networks trained in a purely supervised manner. Regarding the deep learning methods, we sought to identify the best model-loss function combination. Table 2 presents our comparative experimental results for the most important model-loss combinations outlined in Sec. 3.4.

**Table 2.** Model/loss-function comparisons using 4-folds cross validation. Mean of metrics on the test set across folds is reported and standard deviation is in parenthesis. Best of each metric in bold, Statistical Significance of difference in Quality between the two top competing methods is indicated.

Model	${\text{Q}}_{test,\tau =2}$	${\mathrm{Corr}}_{test,\tau =2}$	${\mathrm{Comp}}_{test,\tau =2}$	${\mathrm{PR}}_{test}$
Graph-based	0.7267	0.7884	0.7871	-
unet	0.8230 (0.0348)	0.8583 (0.0417)	0.9529 (0.0165)	0.8535
DRIU [25]	0.8360 (0.0248)	0.8838 (0.0290)	0.9400 (0.0196)	0.8328
i-unet-4	0.8334 (0.0345)	0.8694 (0.0404)	**0.9532** (0.0171)	0.8572
shn-4	0.8464 (0.0263)	0.8877 (0.0333)	0.9484 (0.0209)	**0.8588**
unet-topo	0.8598 (0.0244)	0.9257 (0.0304)	0.9246 (0.0304)	0.8477
shn-4-topo	0.8624 (0.0227)	0.9301 (0.0278)	0.9227 (0.0227)	0.8552
i-unet-4-topo	${\text{0.8671}}^{*}(0.0226)$	**0.9373** (0.0266)	0.9214 (0.0251)	0.8540

The results of extensive paired Wilcoxon significance tests are provided in the appendix. A selection of important significance tests are presented in Tables 2, 3 and 4 where ***, **, and * denote significant differences with p<0.001, p<0.01, and p<0.05, respectively, while ns denotes non significant differences with p≥0.05.

**Table 3.** Effect of iterations (iUNet) and modules (SHN) on quality. Statistically significant differences between top performing model with iterative refinement and top performing model with iterative refinement and topological loss. Best of each model is in bold.

Model	k=2	k=3	k=4	k=5
**i-unet-*k***	0.8302 (0.0335)	0.8322 (0.0330)	0.8334 (0.0340)	0.8312 (0.0317)
**i-unet-*k*-topo**	0.8626 (0.0232)	0.8631 (0.0236)	${\text{0.8671}}^{***}$(0.0226)	0.8661 (0.0210)

**shn-*k***	0.8310 (0.0264)	0.8314 (0.0268)	0.8464 (0.0263)	0.8452 (0.0274)
**shn-*k*-topo**	0.8598 (0.0222)	0.8616 (0.0235)	${\text{0.8624}}^{***}$ (0.0227)	0.8609 (0.0235)

**Table 4.** Effect of base network depth on quality. Statistically significant differences between deeper and shallower base networks are indicated. Best of each model is in bold.

Blocks	unet-topo	i-unet-4-topo	shn-4-topo
5	0.8484 (0.0235)	0.8548 (0.0254)	0.8510 (0.0235)
3	${\text{0.8598}}^{**}$ (0.0244)	${\text{0.8671}}^{***}$ (0.0226)	${\text{0.8624}}^{**}$ (0.0227)

The segmentations achieved by the **unet** constitute a baseline of acceptable quality. The produced vessel maps, however, suffer from subtle topological inaccuracies, such as discontinuous or overly connected branches, and false positives due to image noise or artefacts. This can be attributed to the cross-entropy loss being oblivious to local context around each pixel, in contrast to the perceptual loss which attends to local features creating a complementary learning signal.

Combining the perceptual loss term of (2) with the loss function of (3) significantly boosts performance. Despite not using any form of iterative refinement, **unet-topo** significantly outperforms **unet**, and both iterative and stacked networks that do not make use of this additional loss term. As can be observed in Table 2, the networks that are trained using the perceptual loss show a sharp increase in Correctness values counterbalanced by a slight decrease in Completeness, compared to the same networks trained without it. This leads to improvements in Quality, which translate to smoother and cleaner predictions, albeit missing some very fine details.

Combining both iterative refinement and the topological loss improves performance even further. The model/loss-function combinations that demonstrated the highest performance in terms of Quality were **shn-4-topo** and **i-unet-4-topo**. The difference in performance between **i-unet-4-topo** and **unet-topo** is statistically significant, providing evidence that there exists a synergy between iterative refinement and the incorporation of the topological loss.

According to Table 2, adding iterative refinement (either through stacking or iterations), translates into a concurrent increase of completeness and correctness and therefore of quality.

Table 3 shows that increasing the number of stacked modules or refinement iterations boosts performance, respectively for the SHN and iUNet, with or without the perceptual loss. The optimal number of stacked modules and refinement iteration was 4 while further increasing both to 5 led to slightly worse performance, possibly due to the fact that performance achieved with less refinement steps is already quite high, thus leaving small grounds for improvement. Figure 8 showcases the effect of adding iterative refinement to a model trained with the perceptual loss.

Table 5 presents the cross validated improvements in the Quality metric $\Delta Q^{j+1-j}$ between consecutive refinement iterations j and j+1 for **shn-4-topo** with **i-unet-4-topo**. As observed the second refinement iteration offers a significant boost in performance, while further iterations offer diminishing gains. Using less iterations, however, performs worse overall according to Table 3. Furthermore, as presented in Table 4 using a deeper base network leads to worse results for the top performing networks, a finding that can be attributed to having a limited dataset.

**Table 5.** Improvements through iterative refinement combined with topological loss.

Model	$\Delta Q_{test,\tau =2}^{2-1}$	$\Delta Q_{test,\tau =2}^{3-2}$	$\Delta Q_{test,\tau =2}^{4-3}$
**i-unet-4-topo**	0.01659	0.0016	0.0002
**shn-4-topo**	0.01749	0.0008	<0.0001

Conclusively, **i-unet-4-topo** marginally outperforms **shn-4-topo** (with marginal statistical significance p<0.05) and is also optimal in terms of parameter efficiency, as it requires 1/4 of the parameters of the latter. The fact that the more parameter-heavy **shn-4-topo** is not performing better than the lighter **i-unet-4-topo** can possibly be attributed to the lack of a large training set.

**Fig. 7.**
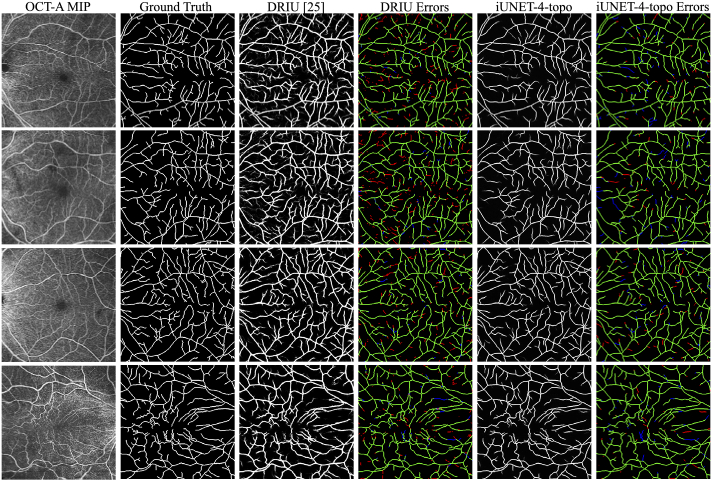
Qualitative comparison of results from Imagenet pretrained and fine-tuned baseline **DRIU** [25] and **i-unet-4-topo**, trained from scratch. The latter was the top performing model/loss function combination. The two methods achieve similar recall. However, **DRIU** exhibits noisier predictions with a considerable amount of false positives. Columns 4 and 6 present centerline errors (dilated by one pixel to improve visibility) made by the two models, with false and true positives shown in red and green respectively, while missed segments are shown in blue.

Finally, **DRIU**, which utilizes pretraining on Imagenet, is significantly outperformed by these two networks trained from scratch. This is not surprising as RGB natural images found in Imagenet differ substantially from grayscale OCT-A images and therefore fine-tuning the pretrained weights offers limited gains in performance. This finding is in-line with the empirical results of [53] that demonstrate very limited gains when using Imagenet weights and architectures for medical imaging tasks, including retinal image pathology grading. A qualitative comparison of **DRIU** and **i-unet-4-topo** can be found in Fig. 7.

**Fig. 8.**
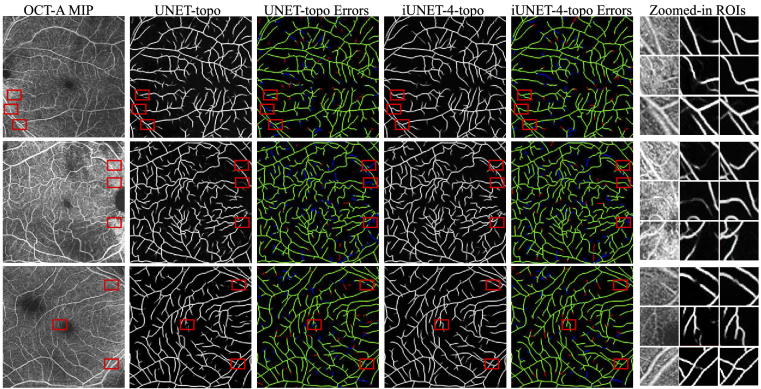
Adding iterative refinement to **unet-topo**: outlined in red are some examples of fine details that are recovered only by **i-unet-4-topo**. The outermost column depicts zoomed-in regions of interest corresponding to the red bounding boxes, and aids with the comparison of the response of the two models. Columns 3 and 5 present centerline errors (dilated by one pixel to improve visibility) made by the two models, with false and true positives shown in red and green respectively, while missed segments are shown in blue.

### 4.2 Dataset size ablation

We evaluated the effect that decreasing training dataset size has on network performance. Specifically, we retrained our best performing network with less data by randomly removing subjects leaving us with 20,10,5 subjects per training set. As in all previous experiments, we used cross-validation and data augmentation while also keeping the test set of each dataset fold the same with previous experiments. This allows us to observe performance decrease solely caused by having less training data. Results for this experiment for **i-unet-4-topo** are presented in Table 6 and indicate that truncating the training set up to 1/3 of the full training set leads to a small but consistent performance decrease, while a considerable drop of almost 4% in performance occurs when training with only 1/6 of the full training set.

**Table 6.** Quality metric when fractions of the full dataset are considered.

Subjects (train set)	5	10	20	30 (full)
**i-unet-4-topo**	0.8334	0.8601	0.8624	0.8671

## 5. Discussion

We present two other possible use-cases of our networks, pretrained on 8mm×8 mm MIPs, on other forms of OCT-A data (3D and 3mm×3 mm scans) without any retraining. We also discuss generalization when using a relatively small dataset.

### 5.1 3D volume segmentation

**Fig. 9.**
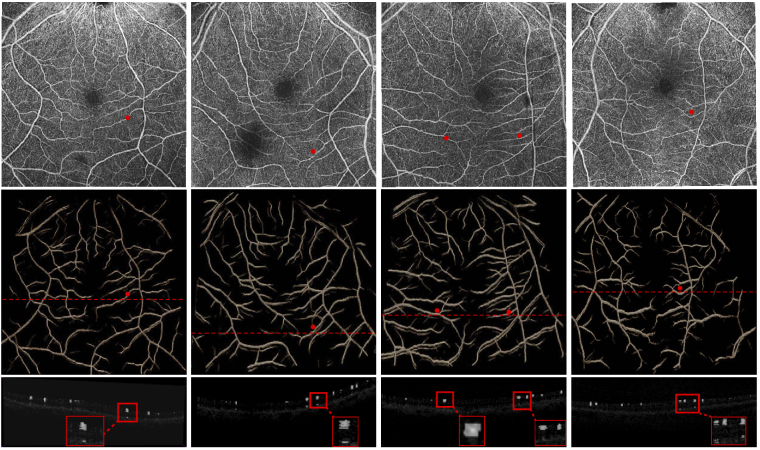
OCT-A 3D segmentation: The 1^st^ row depicts the MIP associated with the raw 3D volume which is per-slice segmented by **shn-4-topo** (the model that gave the best, based on visual inspection, 3D results), with the resulting 3D segmentation displayed below. The 3^rd^ row displays cross-sections of the segmentation (gray) overlayed on the OCT-A cross-section, the location of which is denoted by the red dashed line. Finally zoomed in cross-sectional details are shown (denoted in upper rows by red dots) which reveal the network mistakenly segments shadowing artefacts (1^st^, 2^nd^, 4^th^ columns) below bigger vessels which is normal due to it being unaware of 3D context. A video demonstration of the 3D segmentations is provided as supplementary material.

The raw (non-geometrically flattened) 3D OCT-A volume can be viewed as a sequence of 2D slices. We can obtain a metric 3D segmentation by aggregating 2D per-slice segmentations produced by our models trained on geometrically flattened MIPs. These models, in principle, can generalize to delineating vessels on each 2D slice of the raw unflattened 3D OCT-A, without any retraining. Figure 9, and Visualization 1, depict 3D segmentations obtained with this approach. Due to lack of 3D ground truths the generated 3D segmentation can only be visually evaluated. It is acknowledged that the 3D results are less impressive than the 2D segmentations, for which we provide direct supervision via annotations. However, it is qualitatively demonstrated that our models are able to produce plausible 3D segmentation without ever being provided with any 3D supervision.

### 5.2 Generalization to narrower field of view OCT-A

**Fig. 10.**
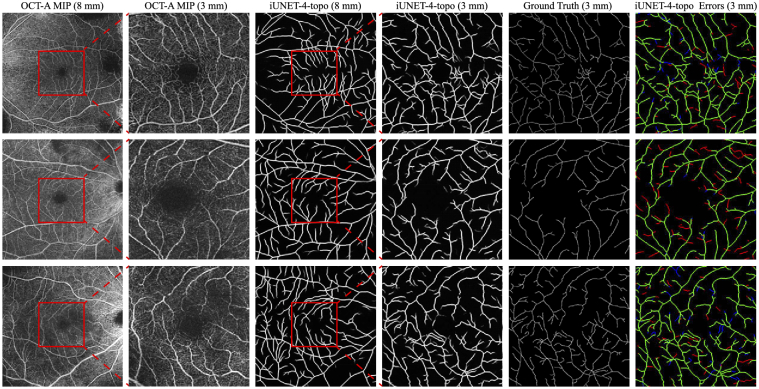
Generalizing to 3mm×3 mm scans: Using **i-unet-4-topo**, we can produce plausible segmentations of the narrower FoV scans which reveal more details of the central part of the macula. The 1^st^ and 2^nd^, and 3^rd^ and 4^th^ columns, demonstrate the correspondence between the two scans and the two segmentations respectively, while the 5^th^ and 6^th^ presents the ground truth centerline and errors with respect to it respectively, with false (red), true (green) positives and missed segments (blue).

All models described in this work were trained using MIPs of 8mm×8 mm OCT-A. We observed these networks can generalize to segmenting 3mm×3 mm FOV OCT-A without retraining. These narrower FOV scans are separately captured scans (rather than digitally zoomed-in versions of wider FoV scans) that focus on details of the center of the macula by trading off size of imaged region. Figure 10 presents qualitative examples accompanied by a comparison with human annotations.

### 5.3 Generalization with limited data and transfer learning

Due to the limited amount of data, in comparison with datasets of natural images, an argument can be raised that training deep networks may lead to over-fitting. Acknowledging this concern we initially experimented with adaptive thresholding techniques that proved ineffective as they constitute purely intensity based methods that are completely unaware of the local geometry of the vessels. Subsequently we formally compared CNNs against a graph-based weakly-supervised method which combines hand-crafted filtering, domain assumptions (such as the tree-like structure of vessels) and simple learning-based classifiers. While this method performed reasonably, it required extensive fine-tuning of its many settings, and was significantly outperformed by even the simpler CNNs. Importantly, the fact that the physical principle and goal of OCT-A as an imaging modality is to highlight vasculature acts as a strong prior embedded into the data. As a result, the task undertaken by the neural network is appropriately solved under a low-data regime. We also addressed this by employing early stopping using a validation set and a wide range of geometric and appearance data augmentation techniques (Sec. 3.3). The latter induce invariance to inter-subject OCT-A variability pertaining to variations in vessel shape or density stemming from the type of the underlying retinal pathology or natural morphological diversity.Significantly, the experiments of Section 4.2 reveal that our top-performing model, aided by extensive online-data augmentation, is able to achieve relatively high performance even when trained on 1/6 of the full training set. Moreover, the usage of the perceptual loss can be interpreted as an alternative form of transfer learning, which typically, consists of fine-tuning a network pretrained, usually, on image classification, on the task of interest. We found that was not optimal for OCT-A vessel delineation as this approach (**DRIU**) was outperformed by networks trained from scratch. Instead, the addition of the perceptual loss, transfers the knowledge embedded in the pretrained network’s feature space, by forcing the predictions and the ground truth to lie close within it. This enables the network to learn to be aware of low to mid level features regarding connectivity and shape in the local neighbourhood of each pixel.

## 6. Conclusion

We presented an effective recurrent CNN for vessel segmentation in OCT-A. Experimentally, we concluded that iterative refinement with weight sharing coupled with a perceptual loss is a well-performing solution to the absence of large amounts of data as it naturally separates the precise curvilinear structure localization into a sequence of increasingly finer delineation steps and leverages a pretrained convolutional network in the form of an auxiliary feature extractor. Our model can extract highly detailed vessel maps from maximum intensity projections of 8mm×8 mm OCT-A scans, and can be reliably utilized even on subjects with vitreo-retinal pathology that causes structural macular abnormalities. Our future work will involve translating these vessel maps in VR surgery through registration to the intraoperative video. We anticipate that our methods can also constitute a first step towards automatically calculating retinal biomarkers, such as vessel tortuosity or density, by providing a binary segmentation of vessels in OCT-A. Our software and trained models will be made available online at https://github.com/RViMLab/BOE2020-OCTA-vessel-segmentation for comparisons and to aid in practical applications. Finally, we plan to make our annotated dataset public, through our collaboration with INSIGHT - the UK’s Health Data Research Hub for Eye Health, which to the best of our knowledge will be the first containing OCT-A scans with human annotated retinal vessels of subjects that underwent vitreo-retinal surgery and more annotated data than current retinal vessel segmentation benchmark datasets [10], [11].

## Figures and Tables

**Fig. 11.**
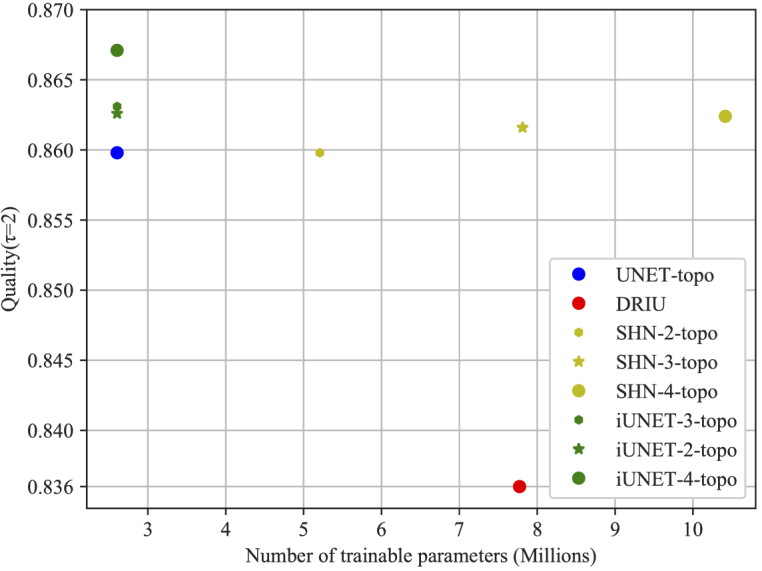
Performance as a function of the number of trainable parameters: **i-unet-4-topo** constitutes the top performing while requiring the minimum number of parameters.

## Appendix

### A.1. Implementation and runtime

All models were implemented within Tensorflow [54] using Python on an NVIDIA Quadro P6000 GPU. Training time varied for different networks and also for training the same network using different data folds, due to the utilization of early stopping according to performance on the validation set. On average, SHN models with 4 modules converged at 2 hour 50 minutes, while iUNet models with 4 iterations converged at 2 hour and 24 minutes. Inference for an input image with a pixel count of 416×416 for both models was 171 ms. Inference time for UNet was 43 ms.

### A.2. Metric 3D OCT/OCT-A data from Zeiss Angioplex

To recover the raw data representing the 3D volume of OCT/OCT-A acquired by Angioplex, a license 266002−1142−523 is required. The extracted data is a series of brightness values assuming isotropic voxels. As isotropy is not the case in OCT/OCT-A acquisitions, the data cannot be used for metric segmentation. Further, the DICOM files that accompany each acquisition are encrypted and obscured. Each OCT/OCT-A acquisition corresponds to multiple DICOM files with occasionally conflicting information. We successfully recovered metric 3D volumes by combining the raw “isotropic” 3D information with an extensive comparison of all DICOM files corresponding to a single acquisition. A series of automated sanity checks and OCT/OCT-A DICOM file comparisons allows the extraction of the width/height/length of the voxel, and the creation of a Nifty volume [55] containing every information required for metric processing of the acquired volumes.

### A.3. Model/loss function comparisons and statistical significance tests

We provide the evaluation metrics of all model/loss function combination in Table 2 and paired statistical tests in Table 7 that indicate the statistical significance of the differences in Quality metric between selected model/loss function combinations where ***, **, and * denote significant differences with p<0.001, p<0.01, and p<0.05, respectively, while ns denotes non significant differences with p≥0.05. Figure 11 shows Quality plotted against the number of trainable parameters.

### A.4. Graph-based baseline

We implemented a graph-based method inspired by state-of-the-art algorithms from [23,24]. The method entails two sequentially applied components.

The first component extracts an over-complete graph $G_{o}$ whose nodes, $V_{o}$, lie on vessels and whose edges, $E_{o}$, roughly constitute a superset of the edges of the ground truth vessel map. To create the graph, the MIP image is filtered using a tubularity filter [15]. Then, the likelihood of a pixel belonging to a vessel centerline is calculated via a sigmoid function that maps the tubularity values to the [0,1] interval. We use a priority queue constructed by the likelihood $p_{i}$ of each pixel i in the image to iteratively select pixels with high tubularity; these form a set S coined seeds. Finally, to construct $G_{o}(V_{o},E_{o})$, we set $V_{o}=S$ and then compute minimum cost paths between pixels of S that are up to a certain distance away from each other. For that, we treat the image as a graph $G(V_{I},E_{I})$ where $V_{I}$ is a set of nodes populated by each pixel on the image and $E_{I}$ is a set of edges that are formed by connecting each node to its 8 nearest neighbors on the image grid. Each such edge is assigned a probabilistic cost [23] that is described by:

(6) $$p_{ij}=d_{ij}\frac{p_{i}log(p_{i})+p_{j}(1-log(p_{j}))}{p_{i}-p_{j}},$$

where i,j indicate pixel indices on the image grid, $d_{ij}$ denotes the euclidean distance between the two pixels and $p_{i}$ is the likelihood that pixel i lies on a vessel centreline.

The second component prunes $G_{o}$ using an SVM classifier to identify paths in $E_{o}$ belong to the true vessel map. Weakly supervised learning is followed to train the classifier, as in [24], using a set of valid and invalid paths that are mined from a collection of over-complete graphs extracted from 20 MIP images. The paths are labeled using heuristic criteria that quantify whether they can be part of the ground truth. From the neighborhood of each path, Histograms of Gradient Deviation descriptors are extracted and encoded to a fixed length descriptor vector using the Bag of Visual Words paradigm. Finally, the paths of $G_{o}$ that are classified as valid, are added to the final vessel map prediction.

**Table 7.** Paired Wilcoxon significance tests for cross validated Quality metric.

Models	unet	DRIU	unet-topo	i-unet-4	i-unet-4-topo	shn-4	shn-4-topo
unet		**	***	**	***	***	***
DRIU	**		***	ns	***	**	***
unet-topo	***	***		***	**	***	ns
i-unet-4	**	ns	***		***	***	***
i-unet-4-topo	***	***	**	***		***	*
shn-4	***	**	***	***	***		***
shn-4-topo	***	***	ns	***	*	***	

**${\text{Q}}_{test,\tau =2}$**	0.8230	0.8360	0.8598	0.8334	0.8671	0.8464	0.8624

### A.5. Ablation study for VGG-feature loss

Networks trained with the loss of (2) (see main text) require a choice of VGG19 feature maps and of their respective weighing factors. Therefore, we train and evaluate **i-unet-4-topo** with equal weighing by factors $\mu_{12}=\mu_{22}=\mu_{34}=10^{-2}$ of the loss terms for $conv_{12},conv_{22},conv_{34}$ and also with unequal weighing factors $\mu_{12}=10^{-2}$, $\mu_{22}=10^{-3}$, $\mu_{34}=10^{-4}$; our hypothesis was that spatially coarser feature maps matter less for segmentation details. We also evaluated dropping $conv_{22},conv_{34}$. As shown in Table 8, using unequal weighing with all 3 feature maps gave slightly higher performance, and thus we employed it in all other experiments.

**Table 8.** Ablation study for the VGG-feature loss on validation sets.

Weighing	Feature Maps	${\text{Q}}_{val,\tau =2}$	${\mathrm{PR}}_{val}$
$10^{-1},10^{-2},10^{-3}$	all	0.8650	0.8520
$10^{-2},10^{-3},10^{-4}$	all	**0.8668**	**0.8538**
$10^{-2}$	all	0.8567	0.8445
$10^{-2}$	only $conv_{12}$	0.8603	0.8534
